# Cross-National Vaccine Concerns and Predictors of Vaccine Hesitancy in Not-Fully Vaccinated Individuals: Findings from USA, Canada, Sweden, and Italy

**DOI:** 10.3390/vaccines10101652

**Published:** 2022-10-01

**Authors:** Rachael Piltch-Loeb, Max Su, Marco Bonetti, Marcia Testa, Eva Stanton, Veronica Toffolutti, Elena Savoia

**Affiliations:** 1 Department of Biostatistics, Harvard TH Chan School of Public Health, Boston, MA 02120, USA; 2 Emergency Preparedness Research Evaluation and Practice (EPREP) Program, Division of Policy Translation and Leadership Development, Harvard TH Chan School of Public Health, Boston, MA 02120, USA; 3 Carlo F. Dondena Research Center and COVID Crisis Lab, Bocconi University, 20136 Milan, Italy; 4 Centre for Evaluation Methods, Wolfson Institute of Population Health, Barts and The London School of Medicine and Dentistry, Queen Mary University of London, London E1 2AB, UK

**Keywords:** COVID-19, vaccine hesitancy, cross-national

## Abstract

Vaccine hesitancy is a key contributor to reduced COVID-19 vaccine uptake and remains a threat to COVID-19 mitigation strategies as many countries are rolling out the campaign for booster shots. The goal of our study is to identify and compare the top vaccine concerns in four countries: Canada, Italy, Sweden, and the USA and how these concerns relate to vaccine hesitancy. While most individuals in these countries are now vaccinated, we expect our results to be helpful in guiding vaccination efforts for additional doses, and more in general for other vaccines in the future. We sought to empirically test whether vaccine related concerns followed similar thematic issues in the four countries included in this study, and then to see how these themes related to vaccine hesitancy using data from a cross-sectional survey conducted in May 2021. We applied CFA and created vaccine concern scales for analysis. We then utilized these results in regression-based modeling to determine how concerns related to vaccine hesitancy and whether there were similar or different concerns by country. The results quantitatively highlight that the same vaccine related concerns permeated multiple countries at the same point in time. This implies that COVID-19 vaccination communications could benefit from global collaboration.

## 1. Introduction

Despite the existence of vaccines that are effective at preventing severe COVID-19 disease and hospitalization, vaccine uptake has stalled in many countries. Vaccine hesitancy is a key contributor to reduced COVID-19 vaccine uptake and remains a threat to COVID-19 mitigation strategies as many countries are rolling out the campaign for booster shots. While the challenge of vaccine hesitancy has received significant attention during the pandemic, it is not unique to COVID-19. Prior to the pandemic, vaccine hesitancy was on the rise, and had been recognized as one of the top ten threats to global health [1].

Globally, there have been efforts to monitor trends in COVID-19 vaccine hesitancy at numerous phases of the vaccination campaign. Notably, research from summer 2020 demonstrated significant variation in global rates of hesitancy before the vaccine was approved. In a seventeen-country study conducted by Rozek et al. from May to June 2020, rates of vaccine hesitancy ranged from 27% to 72% (Russia to Vietnam) [2]. In a nineteen-country study conducted during a similar time frame, Lazarus et al. found that 71% of respondents would be willing to take the vaccine, but also that there was significant heterogeneity in hesitancy rates by country, demographic correlates of hesitancy, and vaccine policy perceptions [3]. Additionally, a forty-country study conducted by Singh et al. during June and July 2020 found a strong association between vaccine hesitancy and misinformation endorsement, and that misinformation endorsement prevalence was higher in low-income regions [4].

In the eighteen months since the first COVID-19 vaccine was administered, national and subnational government agencies have adopted varied communication and policy strategies to drive vaccine promotion in a continuously evolving information ecosystem. Nevertheless, vaccine hesitancy remains a persistent challenge during current vaccination campaign efforts. In a recent systematic review of global vaccine hesitancy rates, Sallam found that relatively high levels of hesitancy (greater than 30%) persisted in several European countries and the USA [5].

The goal of our study is to identify and compare the top vaccine concerns in four countries: Canada, Italy, Sweden, and the USA and how these concerns relate to vaccine hesitancy. While most individuals in these countries are now vaccinated, we expect our results to be helpful in guiding vaccination efforts for additional doses, and more in general for other vaccines in the future. Previous research shows that some of the top COVID-19 vaccine concerns in these countries were related to vaccine safety, speed of vaccine production, ingredients in the vaccine, adverse effects of the vaccine, political and financial gains, and limited perceived risk of COVID-19 [6,7,8,9]. Though the concerns associated with vaccine hesitancy in each of these countries have been examined in a prior study [10], prior research has not looked at such concerns cross-nationally. This study aims to identify which concerns are country specific, and which concerns, instead, appear to affect vaccine hesitancy consistently across multiple countries.

Our review of research conducted at the national level in Canada, Italy, Sweden, and the USA has identified common narratives contributing to vaccine hesitancy within and across these four countries. In the USA, one of the most persistent concerns has been that vaccines cause infertility [11], while in Canada, a study conducted by Griffith et al. found that concerns over the lack of legal liability of vaccine companies contributed to vaccine hesitancy [12]. In Italy, Moscardino et al. found that concerns about the vaccine’s efficacy contributed to vaccine hesitancy among young adults [13]. Studies from both Canada and Sweden have documented concerns regarding vaccine side effects, particularly allergic reactions, as well as about the country of origin of the vaccine [14,15,16]. Further, literature from all four countries demonstrates greater rates of vaccine hesitancy among those who have children than those who do not. Research from all four countries has documented concerns about the safety of COVID-19 vaccines when administered to children, especially those under 12 years of age [11,17,18,19]. A study by the U.S. Centers for Disease Control and Prevention found that these concerns persisted even among parents who were vaccinated themselves [11].

### Specific Objectives

We sought to empirically test whether vaccine related concerns followed similar thematic issues in the four countries included in this study, and then to see how these themes related to vaccine hesitancy. There are three specific objectives of this study:

1. To assess if COVID-19 vaccine related concerns are common across the four countries: USA, Sweden, Italy, and Canada);
2. To identify the relationship between the vaccine related concerns and vaccine hesitancy in each country of interest; and,
3. To identify and interpret heterogeneity in the relationship between vaccine-related concerns and vaccine hesitancy across the four countries.

## 2. Materials and Methods

This study consists of two parts: (1) the development of scales to describe COVID-19 vaccine concerns from a set of nineteen items collected across four countries and (2) analysis of the effect of these concerns on vaccine hesitancy.

### 2.1. Data Collection

We used a cross-sectional study design and collected data using an online survey through the Pollfish mobile platform [20]. The survey was limited to individuals aged ≥18 residing in the USA, Canada, Italy, and Sweden. Each of the four countries shared similarities in their approach to procurement, distribution, and prioritization plans for the vaccines though they also had differences in their implementation and epidemiologic experience [21,22,23,24]. A screening question was used to identify respondents who were not yet vaccinated, or who had received only one dose of a COVID-19 vaccine requiring two doses. The Pollfish platform uses random device engagement (RDE), an approach similar to Random Device Dialing (RDD), to reach users engaged in using mobile applications (rather than calling them) who are identified only by a unique device ID [25]. Like third-party advertising companies, Pollfish pays mobile application developers to display and promote the surveys to their users using crowdsourcing. Pollfish has over 1 billion registered users worldwide. For this survey, a random sample of users who fit the study’s eligibility criteria was initially selected and data were collected between 21–28 May 2021. The study protocol and survey instrument were approved by the Harvard T.H. Chan School of Public Health Institutional Review Board (IRB) on 8 December 2020 (protocol #20-203) and by the Bocconi University IRB on 22 April 2021 (protocol #31146). The respondents were asked to consent to participate in the study immediately before responding to the survey questions. All versions of the questionnaire are provided in the Appendix A and Appendix B to this manuscript. We solicited feedback on the items from a small sample (*n* = 20) of individuals who spoke English, French, and/or Italian to determine if the items we developed measured our intended construct at face value and if the items could be intelligibly translated to French, Swedish and/or Italian. The cognitive debriefing feedback we received regarding item re-wording was incorporated in a revised version of the questionnaire prior to its implementation. The survey was translated into Canadian French and Italian and back-translated into English for validation purposes. All samples had equally distributed quotas by sex and age groups and the Canadian sample was equally distributed between French and English speakers. In Canada, we sampled two different groups, one from the English-speaking part of the country and one from the French-speaking, to fully acknowledge the role that the two different cultures could play in vaccine hesitancy. Canadian respondents were given the option to respond in English or French and the datasets derived from the two samples were analyzed independently. As the minimum amount of time to thoughtfully complete the survey was tested to be three minutes, we used this time criteria as a method for data quality assurance and removed any questionnaire completed in less than three minutes.

### 2.2. Development of the COVID-19 Vaccine Concerns Scales

#### Item Generation Process

Questions related to potential vaccine concerns were created based on a previous analysis of frequently reported tropes and misinformation narratives identified in formative work by the research team and previously published [26]. We conducted an exploratory factor analysis using a principal factor method of factor extraction to determine how the nineteen items of “vaccine information/opinion” could be grouped together to form scales. Before performing the factor analysis, we checked the appropriateness of the assumptions using Bartlett’s test for sphericity and the Kaiser–Meyer–Olkin Measure of Sampling Adequacy. We employed several methods to determine the number of components to retain including (a) eigenvalues above 1, (b) scree plot, (c) factors accounting for at least 5% of the variance and (d) interpretability of factor structure. The internal consistency of the factors formed was checked by Cronbach’s alpha.

The analysis returned three factors. Because the Canadian survey was conducted in English and in French, we created separate groups for the Canadian sample, given that there may be biases associated with combining the two. Therefore, we had five-country language groups in total. In the five country-language groups, we tested the scales for measurement invariance to see if respondents in different country-language groups reacted similarly to the items. To determine measurement invariance across the five country-language groups, we used a multi-group confirmatory factor analysis. The groups were first tested for configural invariance to determine if the same model holds in all groups. If configural invariance holds then metric invariance was tested to see if the factor loadings were the same across groups. If the scales do not show measurement invariance using the confirmatory factor analysis method, we would further explore measurement invariance using a differential item functioning (DIF) analysis. DIF occurs when the probability of endorsing an item is not the same in different groups after controlling for the level of the trait being measured. We performed a DIF analysis by fitting an ordinal logistic regression model (1, 2) to each item with the subject’s trait score generated from an item response theory (IRT) model called the grade response model (GRM) as a covariate. To test for nonuniform DIF, we used a likelihood ratio test (LRT) and the change in R2 or effect size between the model with and without the interaction between country-language groups and the subject’s trait score in the model. We required both LRT *p*-values to be less than the Bonferroni adjusted 0.003 (0.05/17) and a change in R2 of at least 0.035 [27] to classify an item as having nonuniform DIF. For uniform DIF we required the *p*-value associated with the group variable to be significant at the 0.001 level and the absolute value of the log odds ratio to be greater than 0.64 [28,29].

### 2.3. Concerns about Contracting COVID-19 (“CCC”) Scale

In addition to the vaccine concerns items, the questionnaire contained three questions asking the respondent about their concerns about contracting COVID-19 and infecting others. These questions were analyzed with factor analysis to see if they could be combined into a single latent scale. Bartlett’s test for sphericity had a *p* < 0.001 and the Kaiser–Meyer–Olkin Measure of Sampling Adequacy was equal to 0.7, which suggested a factor analysis of the data to be a reasonable approach. A principal component factor analysis of the data found one eigenvalue to be greater than one and the scree plot indicated that a single factor should be retained, indicating the items reflect a latent construct. The three-factor loadings were reasonably large at 0.70, 0.70 and 0.75 and Cronbach’s alpha for the three items was 0.79, indicating good internal consistency. Thus, these results showed that the three items measure a unidimensional construct, which we name “Concerns about contracting COVID-19”, or CCC. The CCC scale was created as the sum of the three ordinal items and ranged from 0 to 6 for least to most concerned about contracting COVID-19.

### 2.4. COVID-19 Vaccine Hesitancy Outcome Measure

The primary outcome was the likelihood of the respondent taking the COVID-19 vaccine and was assessed by the question, “If you were offered a COVID-19 vaccine—at no cost to you—how likely are you to take it?” The response categories (with percent choosing each response) were “Very likely” (52%), “Somewhat likely” (18%), “I am not sure” (12%), “Somewhat unlikely” (4%), “Very unlikely” (9%) and “I would not take it at the moment but would consider it later on” (5%). To avoid small cell counts and unstable model parameter estimates, we recoded the variable into three categories by combining the “I would not take it” and “Very unlikely” responses to create a low likelihood to vaccinate category (“Very unlikely” or “1” below); combining “Somewhat unlikely”, “I am not sure” and “Somewhat likely” to create an unsure or middle likelihood category(“Unsure” or “2” below); and the “Very likely” response made up the high likelihood category (“Very likely” or “3” below).

### 2.5. Statistical Analysis

In order to determine the effect of each of the three factor scales on vaccine hesitancy, we fit ordinal logistic regression models. We first tested whether each factor scale had a linear relationship with the outcome by including a quadratic term for each factor scale in the model. If the quadratic term was significant, we would use four category variables that represent the quartiles for the scale. While the three-factor scales were the main predictors of interest, we were also interested in whether the effect was the same across the five country-language groups. As such, when the interaction between country-language groups and a factor score was significant at the 0.05 level, it was included in the model, and we would investigate the reason for the interaction by testing contrasts between the country-language group and the factor score at the 0.05 level. In our analysis, we adjusted for country-language groups, age, gender, education, income, vaccine availability, indicator of prior refusal of taking recommended vaccines, and the CCC scale. The proportional odds assumption for the ordinal logistic model was tested using the Brant test and if it failed, we would fit a partial proportional odds model instead.

## 3. Results

### 3.1. Sample Characteristics

We gathered responses from 3663 individuals living in Canada (*n* = 985), Italy (*n* = 986), Sweden (*n* = 965) and the USA (*n* = 727). The Canadian respondents were further divided into 493 and 492 individuals responding in English and French, respectively. Due to the Pollfish sampling method, the total sample of 3663 subjects and the sample from each country-language population was almost perfectly evenly divided among the five age categories see (Table A1). Likewise, the sample was evenly split between males and females. For education in the total sample, 7% had less than a high school education, 36% had a high school degree, 19% had some college, 23% had a bachelor’s degree, 13% had post-graduate degree and 1% had “other” as a level of education. Differences in education level across the groups found the US sample had a higher number of individuals with a postgraduate degree (30%) and a lower number for high school (22%) while Sweden and Italy had a higher percentage with high school at 48% and 45%, respectively. English and French Canadians had higher percentages with some colleges at 27% and 29%, respectively and English Canadians also had higher percentages of bachelor’s degrees at 36%. In the total sample, 77% of the respondents stated that they were eligible to receive a COVID-19 vaccine. This number ranged from 64% in Sweden to 85% among English Canadians. Overall, 25% of respondents said that in the past they had not taken a vaccine that had been recommended by a healthcare provider, with the US population having the highest number at 48% and Italy having the lowest at 9%. The CCC scale had a mean of 3.2 and a standard deviation of 1.9 in the total sample, with French Canadians having the lowest mean value at 3.0 and Italians having the highest mean value at 3.8.

### 3.2. Objective 1: Vaccine-Related Concerns by Country

To determine if there were similar underlying concerns by country, we used factor analysis of the vaccine-specific items. We then used the results of the factor analysis to create a scale that was used in subsequent models.

#### 3.2.1. Exploratory Factor Analysis

With 3663 subjects our sample size was sufficiently large for conducting an exploratory factor analysis, whose appropriateness was confirmed by Bartlett’s test for sphericity (*p* < 0.001) and the Kaiser–Meyer–Olkin Measure of Sampling Adequacy (KMO = 0.941) [30]. For determining the number of factors to retain, two eigenvalues were above 1 while the scree plot and 5% rule suggested that three factors should be retained. Because of this, we fit the factor analysis by specifying between one and three-factor solutions and reviewed the results to help determine the factor solution to retain. For the two- and three-factor models the oblique promax rotation was used because many of the items had moderate to high correlation with each other. Two items were removed from the analysis because they either had low factor loadings (less than 0.4 on all factors) or a high factor cross-loading (above 0.4). The three-factor solution was chosen see (Table 1) due to the relative clarity of its factor structure and its conceptual interpretability. The correlation between factors 1 and 2 was high at 0.70, while correlations between factor 1 and 3 and between factor 2 and 3 were equal to 0.21 and 0.09, respectively. We name the three factors representing three constructs: “Vaccine effects”, “Vaccine value” and “Societal freedoms”.

Internal consistency as measured by Cronbach’s alpha was 0.84, 0.87 and 0.63 for factors 1, 2 and 3, respectively.

#### 3.2.2. Measurement Invariance Using a Confirmatory Factor Analysis (CFA)

In examining whether the same factor structure (configural invariance) held across the five country-language groups, we fit the three-factor CFA model to the data for each group, and tested model fit using root mean square error of approximation (RMSEA), the comparative fit index (CFI) and Tucker–Lewis Index (TLI). For each country, the RMSEA was less than 0.07 which was less than the 0.08 threshold used to identify models that fit the data reasonably well. The CFI and TLI were above 0.9 except for Sweden (0.89) which also indicates adequate fit to the data see (Table 2).

Given that the configural invariance showed the same model structure fit the data for each country reasonably well, we then tested metric invariance or whether the factor loadings were the same across countries. Each factor failed the test of metric invariance across the five country-language groupings with $X_{24}^{2}\;$ = 47.2 (*p* = 0.0032), $X_{20\;}^{2}$ = 54.2 (*p* = 0.0001) and $X_{12\;}^{2}$ = 23.7 (*p* = 0.0221) for Factors 1, 2 and 3, respectively.

#### 3.2.3. Differential Item Functioning (DIF) Analysis

Since metric invariance did not hold across country-language groups, we further investigated differences among groups in the functioning of items using a DIF analysis. Factor 1 did not show DIF across country-language groups. We found uniform DIF in one item (vax_op_7_microchip) from factor 2 and one item (vax_op_4_hc_share_op) from factor 3. We created subject scores for the three factors from the latent ability estimates of a GRM. For factors 2 and 3, the model was fit with only items that had no DIF between country-language groups (Table 3).

### 3.3. Objective 2: Vaccine-Related Hesitancy by Country

#### 3.3.1. Modeling of the Vaccine Hesitancy Outcome

The interpretation of the results of this analysis can be best summarized through the predicted probabilities of the three levels of the outcome variable (1 = Very unlikely; 2 = Unsure; and 3 = Very likely), by country and by quartiles of the factors. In addition, one may examine the odds ratios of the two logits defined by the model, to quantify the differences in the odds across the different quartiles of the factors. Below we summarize the main findings and refer the reader to Appendix A for a detailed analysis of the individual effects, and, in particular, of the interaction terms with the country-language groups (for the Vaccine Value Factor and Societal Freedoms Factor).

#### 3.3.2. Model Main Effects

**Figure 1:**
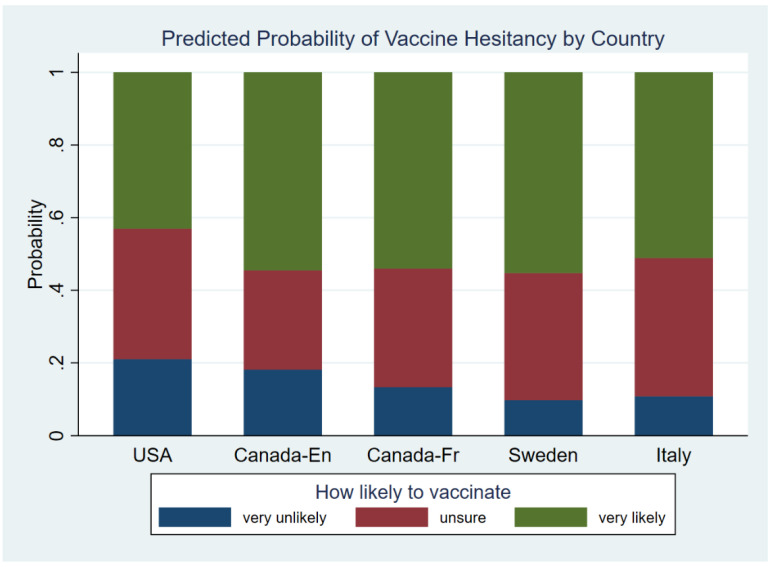
Predicted Probability of Vaccine Hesitancy by Country.

Although the model had significant interactions between country-language groups and factors 2 and 3, we first look at results for the main effect of country-language group which will help in the interpretation of these interactions. The model showed that the USA group had significantly higher odds of selecting the “very unlikely” outcome compared to English Canadians (OR = 1.8, *p* = 0.001), French Canadians (OR = 2.2, *p* < 0.001), Swedes (OR = 4.4, *p* < 0.001) and Italians (OR = 3.8, *p* < 0.001), while also having significantly lower odds of choosing “very likely” to vaccinate compared to English Canadians (OR = 0.45, *p* < 0.001), French Canadians (OR = 0.50, *p* < 0.001), Swedes (OR = 0.45, *p* < 0.001) and Italians (OR = 0.59, *p* = 0.001). English Canadian respondents had higher odds of selecting “very unlikely” to vaccinate compared to Swedes (OR = 2.4, *p* < 0.001) and Italians (OR = 2.1, *p* = 0.003) and French Canadians had higher odds of selecting “very unlikely” to vaccinate compared to Swedes (OR = 2.0, *p* = 0.006) and Italians (OR = 1.7, *p* = 0.035). Results shown in Figure 1. 

### 3.4. Objective 3: Vaccine Related Concerns Effect on Vaccine Hesitancy including Exploration of Heterogeneity

#### 3.4.1. Vaccine Effects Factor

**Figure 2:**
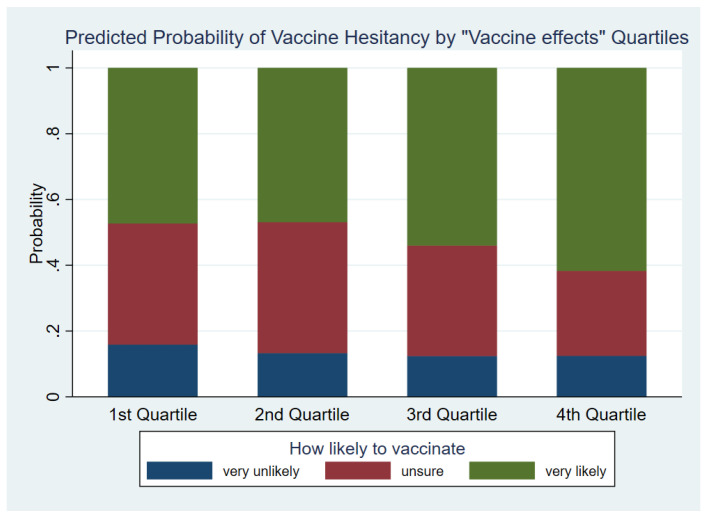
Predicted Probability of Vaccine Hesitancy by “Vaccine effects” Quartiles.

The main effect for the Vaccine effects factor was a significant predictors of vaccine hesitancy with $X_{6}^{2\;}$ = 55.94 (*p* < 0.001). Looking at contrasts between quartiles of the Vaccine effects factor showed that individuals in Q2 and Q3 had significantly higher odds of choosing the “unsure or very likely” to vaccinate category compared to people in Q1, with OR (*p*-value) equal to 1.4 (0.025) and 1.5 (<0.001), respectively. Equivalently, subjects in Q1 had 1.4 and 1.5 times the odds of selecting the “very unlikely” to vaccinate outcome compared to Q2 and Q3, respectively. For each comparison between people in a higher vs. lower quartile of the Vaccine effects factor, the odds of selecting “very likely” to vaccinate was significantly greater than one all (*p* < 0.001) with the exception of the contrast between quartiles 1 and 2. This effect ranged from an OR of 1.5 for Q3 vs. Q1 to an OR of 2.6 for Q4 vs. Q2. The interaction between the vaccine effects factor and country-language group was not significant ($X_{6}^{2\;}$ = 31.53, *p* = 0.139) and was dropped from the model. Results shown in Figure 2. 

#### 3.4.2. Vaccine Value Factor

**Figure 3:**
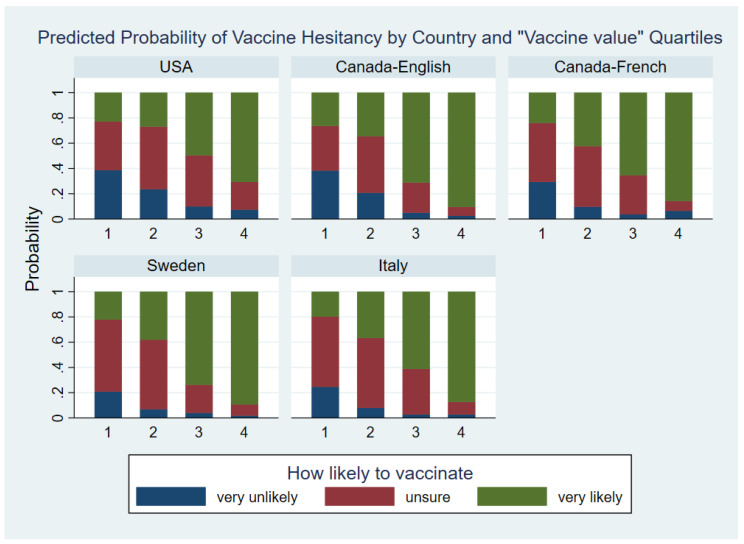
Predicted Probability of Vaccine Hesitancy by Country and “Vaccine value” Quartiles.

For the “Vaccine value” factor 2, in the total sample and within each country-language group higher levels of this factor were associated with a more likely to vaccinate outcome, while the magnitude of this association varied by country-language groups see (Table A2 and Figure 3). The interaction between factor 2 and country-language group was significant with $X_{24}^{2\;}$ = 39.03, *p* = 0.0271. The analysis of r differences between country-language groups in the effect of factor 2 quartiles and by country differences are summarized in Appendix A. Overall, differences between the country-language groups in the effect of the Vaccine value factor were mainly caused by relatively smaller effects in the USA group for both logits and a larger effect in the English-Canadian group for logit A.

#### 3.4.3. Societal Freedoms Factor

**Figure 4:**
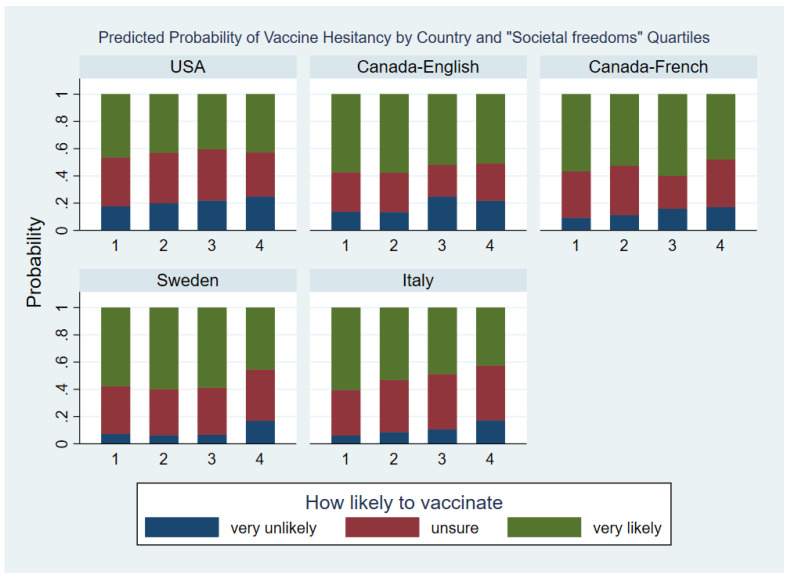
Predicted Probability of Vaccine Hesitancy by Country and “Societal freedoms” Quartiles.

Lastly, let us turn to Factor 3 (“Societal freedoms”). In the total sample and within each country-language group, higher levels of this factor tended to be associated with a lower willingness to vaccinate, but the magnitude of this association varied by country-language groups see (Table A2 and Figure 4). Factor 3 “Societal freedoms” -by-country-language group interaction was significant with $X_{24}^{2\;}$ = 46.31 (*p* = 0.004). In general, the difference in the effect of The Societal freedoms factor across the country-language groups was due to the following causes.

First, the effect of Societal freedoms factor in the USA population tended to be small with OR between quartiles mostly being close to one for both logits. Secondly, in the English Canadian group the effect of Societal freedoms factor was small when comparing Q1 to Q2 and Q3 to Q4 but relatively large between Q2 and Q3. Lastly, in the Swedish population the effect of Societal freedoms factor was similar across Q1 to Q3 while those in Q4 tended to be less likely to vaccinate.

## 4. Discussion

The purpose of this study was to examine whether vaccine related concerns followed similar thematic issues in four countries (US, Canada, Sweden and Italy), and also to determine how these themes related to vaccine hesitancy. This analysis was articulated into three objectives. Below we discuss the results presented in the context of these three objectives.

The first objective was to empirically test if there were common COVID-19 vaccine-related concerns in four countries: USA, Sweden, Italy, and Canada during the initial phase of the vaccine rollout (May 2021). Ultimately, seventeen of the nineteen items were retained into three factors. Each factor reflected a different vaccine-related theme, vaccine effects, vaccine value, and societal freedoms. Vaccine effects capture concerns and perceptions related to the contents of the vaccine, its production, and possible side effects. Vaccine value captures concerns related to the usefulness of the vaccine on a population level like whether or not the vaccine helps to protect oneself or others. Societal freedoms capture concerns related to what will and will not be allowed based on vaccination status including freedom of movement, freedom of vaccine choice, and freedom of speech related to the vaccine itself. Though some of these specific survey items have been identified in the literature from a single country [13,14,15,31,32,33,34] this is the first analysis to identify that these concerns were common in multiple countries at the same time. This demonstrates that concerns are not limited to one country, but rather spread across borders. An explanation may be that the information ecosystem has no borders, and that the use and prevalence of social media across these countries enables rapid proliferation and spreading of information, both factual information and mis-and disinformation [9,35,36]. The perception of the vaccine is largely tied to the content that individuals see and hear. Having shared concerns on the perception of the vaccine cross-nationally demonstrates how distributed these vaccine related concerns are, despite different vaccination policies (i.e., mandatory versus voluntarily) and public health systems across these countries.

The second objective of this analysis was to identify the relationship between the vaccine-related concerns and vaccine hesitancy in each country of interest. We found that Vaccine effects (Factor 1) was a significant predictor of vaccine hesitancy ($X_{6}^{2\;}\;$ = 55.94, *p* < 0.001). Those who had the lowest quartile of the Factor 1 scores, meaning had the lowest level of concern about this construct, were more hesitant (likely to indicate they were unsure or very unlikely to get vaccinated) compared to those in higher quartiles on the factor (especially those in Q2 and Q3). Looking at Vaccine values (Factor 2), higher levels of the Vaccine value factor were associated with a higher likelihood of intent to get vaccinated. Those who were in the third and fourth quartiles of the factor (meaning they perceived the vaccine to have higher value to themselves and others) were more likely to be willing to get the vaccine compared to those in lower quartiles. For Societal freedoms (Factor 3), higher levels of the factor tended to be associated with a lower willingness to vaccinate, meaning those who were more concerned about the freedoms that had been linked to the vaccine, were less likely to accept the vaccine. Examining the graphs presented in the Section 3, we can see that increases in perception of positive vaccine effects led to increases in the probability a subject would be in the very likely to vaccinate category compared to the unsure category. While examining vaccine values, the trend shows that increases in perception of vaccine values are associated with higher likelihood of vaccination compared to the other two categories, there is variation by country. The effect of the vaccine value factor appears to be larger than the other 2 factors (regardless of country). In general, as societal freedom concerns increase, subjects are less likely to be willing to get the vaccine, although this differs by country.

Finally, the third objective was to identify heterogeneity across the countries in the relationship between vaccine-related concerns and vaccine hesitancy. We found no variation by country in the relationship between vaccine effects and vaccine hesitancy, which suggests that there was a consistent relationship between this theme and vaccine hesitancy across countries. The Vaccine effects factor may reflect exposure to misinformation narratives—including issues related to vaccine contents, fertility, impact on DNA, etc. that emerged related to the vaccine [14,15,18,37]. The consistency in relationship between this factor and hesitancy, suggests that these themes were circulating across all four countries, and implies that these narratives may have been the ones to focus on globally in the fight against misinformation.

In contrast, we found there to be between-country differences in the effect of both Vaccine value and Societal freedoms on vaccine hesitancy. Overall, differences among the country-language groups in the effect of the Vaccine value factor on hesitancy was mainly caused by relatively smaller effects in the USA group and a larger effect in the English-Canadian group. To contextualize our findings, we explored the epidemiologic context of the virus and status of vaccination of the population across all four countries and within Canada during the study time period (note, all data referenced is from 15 May 2021). The US had the lowest number of daily cases as measured by cases per million followed by Italy, Canada, and Sweden (101.14, 121.67, 158.49, and 400.46 respectively); while Italy had the highest case fatality rate followed by Canada, the US, and Sweden (2.99, 1.87, 1.76, and 1.38 respectively) [38]. At the time of the study, all adults over the age of twelve were vaccine eligible in the US and Canada, while in Italy (though there was regional variation), most adults over 40 were vaccine eligible, and in Sweden, most adults over 50 were vaccine eligible [39,40,41,42]. Within Canada, there were mixed results on vaccine uptake among French and English-speaking provinces, but one study found that French speakers in Quebec were more likely to be vaccine hesitant [43,44]. This variation in context in each country may mean that the actual perceived value of the vaccine was different in different places; for example, the comparably lower number of new cases per day in the US, as the vaccine campaign began or the virus was leveling off, may have suggested, the value to each individual of getting vaccinated during the study period was actually lower.

The value of the vaccine may also have been discussed differently cross-nationally. In the US, messaging about the value of the vaccine was varied and highly politicized, ranging from a “patriotic duty”, and a vital tool to save lives, to narratives portraying the vaccine as of limited value [45,46]. In contrast, Canadian government messaging about the seriousness of COVID-19 has been largely consistent, and the vaccine has been promoted as the nation’s path to normalcy [47]. These mass communication campaigns get picked up on social media and in popular press and can shift perception of the vaccine. It is not possible to distinguish the specific drivers of the country differences on perceived vaccine values, but we hypothesize there is an effect of both the epidemiologic context, experience with the virus, and perceptions of authority and vaccination.

In terms of Societal freedoms, there were also differential effects across countries. The effect of the Societal freedoms factor in the US population tended to be small overall, which was especially in contrast to the English-Canadian group where there was greater variation between the two middle quartiles (Q2 and Q3), and the Swedish population where there was a larger effect for those in the highest quartile (Q4), who tended to be less likely to vaccinate. These differences may be best contextualized by in-country efforts at the time of the study. Vaccination efforts in Canada and the US were strongly tied to reopening policies. However, unlike Canada and the USA, where government messaging was initially optimistic and promoted the vaccine as a tool to end the pandemic, in Sweden government messaging was more conservative about how the vaccine would affect the country’s ability to reopen, warning Swedes that it would be a “long process” [48].

## 5. Limitations

There are limitations to this study. First, the samples from each country are not representative of the country-wide population. Rather we make between country comparisons of a population that has been sampled in the same way, and therefore our analyses are only focused on what we see in these comparisons rather than broader population-wide conclusions. Further, in our outcome we are looking at intentions to vaccinations rather than actual vaccinations. Finally, these results represent one snapshot in time. The prevalence and support of the factors identified here likely has evolved over time, and further investigation is needed to understand the ongoing salience of these issues. Findings may not be generalizable beyond the countries described here.

## 6. Implications

The implications of this study are multi-faceted. Though there is evidence from social media analyses that there were similar cross-national concerns related to COVID-19 vaccination, this study quantitatively highlights that the same vaccine related concerns permeated multiple countries at the same point in time. This implies that COVID-19 vaccination communications could benefit from global collaboration, at least in the countries analyzed here. Early identification of narratives of concern- such as those related to the development and effect of the vaccine could be a particular focus area for collaborative efforts. Additionally, all of the three themes we identified as having an effect on vaccine hesitancy in May 2020, are still prevalent online in more recent analyses of online and offline narratives [13,14,49]. Given that vaccine hesitancy is still an issue in many countries rolling out booster shots, the relationships identified here, and the relative importance of each of these factors in each country, can be helpful in the development of communication efforts on the narratives that seem to have the greatest effect in that country.

## Figures and Tables

**Table 1:** Factor Loadings, item-test correlation, and communalities.

Item	Factor Loading	Item-Total Correlation	Communality
Factor 1 “Vaccine effects”			
misin_1_c19_vax	0.54	0.68	0.36
misin_2_tox_ingred	0.65	0.76	0.51
misin_3_DNA	0.56	0.73	0.45
misin_4_infert	0.63	0.73	0.45
misin_5_other_dis	0.68	0.73	0.45
misin_6_fast_prod	0.54	0.69	0.39
misin_7_rights	0.47	0.69	0.38
Factor 2 “Vaccine value”			
vax_conf_1_work	0.76	0.82	0.64
vax_conf_2_friends	0.84	0.84	0.67
vax_conf_3_herd	0.86	0.85	0.7
vax_conf_4_c19worse	0.61	0.76	0.46
vax_conf_5_natural	0.51	0.71	0.38
vax_op_7_microchip	0.49	0.71	0.43
Factor 3 “Societal freedoms”			
vax_op_1_decide	0.59	0.72	0.36
vax_op_2_brand	0.57	0.72	0.33
vax_op_3_no_restrict	0.41	0.63	0.23
vax_op_4_hc_share_op	0.55	0.69	0.33

**Table 2:** Three factor model goodness-of-fit measures by country-language.

Country-Language	RMSEA	CFI	TLI
USA	0.053	0.957	0.950
Canada-English	0.058	0.937	0.926
Canada-French	0.068	0.939	0.928
Sweden	0.069	0.902	0.885
Italy	0.063	0.915	0.900

**Table 3:** Estimated Person Ability Scores by Country-language groups with no DIF items.

Country-Language	Vaccine Effects (Factor 1)	Vaccine Value (Factor 2)	Societal Freedoms (Factor 3)
USA	−0.18 ± 1.13	−0.39 ± 0.98	0.02 ± 0.70
Canada-English	0.19 ± 0.99	0.13 ± 0.92	−0.18 ± 0.64
Canada-French	0.15 ± 1.01	0.15 ± 1.02	0.09 ± 0.86
Sweden	−0.12 ± 0.82	−0.03 ± 0.89	−0.05 ± 0.83
Italy	0.08 ± 0.76	0.17 ± 0.85	0.08 ± 0.82

## Data Availability

Data is available upon request.

## Appendix A. Methods

### Appendix A.1. Modeling Vaccine Hesitancy Outcome

We analyzed the linearity of the relationship between the outcome and each factor score in the ordinal regression model and found that the quadratic term was significant for each factor score all (*p* < 0.001). Instead of using quadratic predictors, to allow flexibility in the relationship we categorized each factor score into four groups based on the quartiles of the factor score in the total sample and used these in the final analysis see (Table A1). Next, the Brant test of the proportional odds assumption for the model with the three factor scores and all covariates rejected the assumption ($X_{33}^{2\;}$ = 182.2, *p* < 0.001) so the final model was fit using a partial proportional odds model. In this model the three levels of the outcome were fit using two logit functions with the first one for the log odds of “unsure or very likely to vaccinate” versus “very unlikely” (“2 + 3 vs. 1”) which we call logit A. The second logit is for the log odds of “very likely to vaccinate” versus “unsure or very unlikely to vaccinate” (“3 vs. 1 + 2”), which we call logit B.

### Appendix A.2. Vaccine Value Factor Interaction Term

For comparisons between subjects in Q2 versus Q1, the odds ratio between being in the unsure or very likely versus very unlikely to vaccinate (logit A) in the USA group (OR = 2.3) was about half the odds ratio in the French Canadian (OR = 4.8), Swedish (OR = 4.4) and Italian group (OR = 4.8) with all *p*-values for the ratio of ORs between USA and other country-language groups less than 0.05. Likewise, the OR between very likely versus very unlikely or unsure (logit B) in the USA (OR = 1.3) was significantly less than the French Canadian (OR = 2.6), Swedish (OR = 2.4) and Italian group (OR = 2.6) resulting in ratio of ORs equal to 0.5 for each comparison and all *p* < 0.05. Between Q3 and Q1, the OR for logit A and logit B in the USA were significantly less than the Canadian English and Italian groups with the ratio of OR equal to 0.4 and 0.5, respectively and both *p* < 0.05. In addition, for logit B, the OR for the USA was 0.3 times the OR for Sweden with *p* = 0.0002. Comparing Q4 to Q1, the OR in the USA for logits A and B was 0.3 the OR for English Canadians (*p* = 0.0265). For logit B the OR in the USA was 0.2 times the OR in Sweden and Italy (both *p* < 0.05). Within Canada, the OR of logit 1 for English-speaking respondents was 5.0 times that of the French speakers with *p* = 0.0173. Comparing Q3 to Q2, the logit A OR for English Canadians was 3.3 times greater than Sweden (*p* = 0.0296). For logit 2 the OR in the USA and Italy was significantly less than the OR in Sweden with the ratio of OR equal to 0.5 for both and *p*-values of 0.0420 and 0.0314, respectively. In comparisons between Q4 and Q2 the OR in the English Canadian group for logit 1 was 8.4 and 4.1 times larger than French Canadians and Italians, respectively with both *p* < 0.05. Comparing Q4 to Q3, the OR for English Canadians for logit A was 4.3 times that of French Canadians (*p* = 0.0376) and the OR for French Canadians was 0.2 times the Swedish group (*p* = 0.0466).

### Appendix A.3. Societal Freedom Interaction Term

This interaction was driven by particular differences in the effect of the Societal freedoms factor between country-language groups which are shown in the Appendix A and Appendix B. For logits A and B the OR for comparing Q2 versus Q1 of factor 3 in the Swedish population was 2.0 times greater than the OR in the Italian population (*p* = 0.0374). In comparing Q3 to Q1 of Societal freedoms factor, the OR for both logits in the Swedish group was 2.4 times that of the Italian group (*p* = 0.0079). For logit 1 the OR for English Canadians was 0.3 times that of Swedes (*p* = 0.0055) and for logit 2 the OR for French Canadians was 2.8 times that of Italians (*p* = 0.0186). Comparing Q4 to Q1, the OR for both logits in the USA group was 2.0 times greater than the OR in the Swedish group (*p* = 0.0269) and 2.7 time that of the Italian group (*p* = 0.0020). Comparing Q3 to Q2, the OR for logit A in the English Canadian group was 0.4, 0.3 and 0.4 time the USA, Swedish and Italian groups, respectively with all *p* < 0.05. Comparing Q4 with Q2, the OR for both logits in the USA was 2.9 times greater than Sweden (*p* = 0.0008) and 2.0 times higher than Italy (*p* = 0.0226) and French Canadians were 2.2 times higher than Swedes (*p* = 0.0302). Comparing Q4 to Q3, the OR for both logits in the USA was 3.2 times that of Sweden (*p* = 0.0004). For logit A the English Canadian OR was 5.3 times greater than Sweden (*p* = 0.0004) and 3.0 times larger than Italy (*p* = 0.0151). For logit B2 the OR in the USA was 2.6 times larger than for French Canadians (*p* = 0.0214) and the English Canadians were 2.6 times larger than the Swedes (*p* = 0.0326).

**Table A1:** Demographics and Subject Characteristics.

	USA *n* (%)	Canada English *n* (%)	Canad French *n* (%)	Sweden *n* (%)	Italy *n* (%)	Total
Age						
18–24	142 (20)	99 (20)	99 (20)	188 (19)	194 (20)	722 (20)
25–34	147 (20)	95 (19)	98 (20)	195 (20)	196 (20)	731 (20)
35–44	143 (20)	99 (20)	97 (20)	195 (20)	198 (20)	732 (20)
45–54	149 (21)	100 (20)	99 (20)	192 (20)	198 (20)	738 (20)
55+	146 (20)	100 (20)	99 (20)	195 (20)	200 (20)	740 (20)
Gender						
Male	364 (50)	246 (50)	245 (50)	477 (49)	489 (50)	1821 (50)
Female	363 (50)	247 (50)	247 (50)	488 (51)	497 (50)	1842 (50)
Education						
<High school	49 (7)	20 (4)	29 (6)	89 (9)	83 (8)	270 (7)
High school	162 (22)	83 (17)	155 (32)	463 (48)	440 (45)	1303 (36)
Some college	140 (19)	131 (27)	145 (29)	161 (17)	133 (13)	710 (19)
Bachelor’s degree	132 (18)	177 (36)	119 (24)	155 (16)	277 (28)	860 (23)
Post-graduate degree	221 (30)	74 (15)	44 (9)	85 (9)	51 (5)	475 (13)
Other	22 (3)	8 (2)	0 (0)	12 (1)	2 (0)	44 (1)
Income						
Prefer not to say	52 (7)	52 (11)	56 (11)	156 (16)	196 (20)	512 (14)
Lower I	141 (19)	53 (11)	63 (13)	118 (12)	206 (21)	581 (16)
Lower II	108 (15)	71 (14)	78 (16)	90 (9)	303 (31)	650 (18)
Middle I	93 (13)	102 (21)	82 (17)	95 (10)	155 (16)	527 (14)
Middle II	85 (12)	87 (18)	79 (16)	114 (12)	82 (8)	447 (12)
High I	38 (5)	76 (15)	76 (15)	91 (9)	29 (3)	310 (8)
High II	73 (10)	27 (5)	37 (8)	90 (9)	6 (1)	233 (6)
High III	137 (19)	25 (5)	21 (4)	211 (22)	9 (1)	403 (11)
Are you eligible to receive a COVID-19 vaccine?						
No	176 (24)	73 (15)	93 (19)	343 (36)	153 (16)	838 (23)
Yes	551 (76)	420 (85)	399 (81)	622 (64)	833 (84)	2825 (77)
In your life, were you ever recommended a vaccine (other than the COVID-19 vaccine) by a healthcare provider that you did not take?						
No	375 (52)	391 (79)	382 (78)	691 (72)	893 (91)	2732 (75)
Yes	352 (48)	102 (21)	110 (22)	274 (28)	93 (9)	931 (25)
	mean ± SD	mean ± SD	mean ± SD	mean ± SD	mean ± SD	mean ± SD
Concerns about contracting COVID-19 score	3.3 ± 2.1	3.3 ± 1.8	3.0 ± 12.0	3.5 ± 1.8	3.8 ± 1.7	3.2 ± 1.9

Income levels varied by country. In the USA Lower I = Under $25,000, Lower II = $25,000–$49,999, Middle I = $50,000–$74,999, Middle II = $75,000–$99,999, High I = $100,000–$124,999, High II = $125,000–$149,999, High III = $150,000+. In Canada Lower I = Under $15,000, Lower II = $15,000–$29,999, Middle I = $30,000–$44,999, Middle II = $45,000–$74,999, High I = $75,000–$124,999, High II = $125,000–$199,999, High III = $200,000+. In Sweden Lower I = Under 90,000 kr, Lower II = 90,000 kr–179,999 kr, Middle I = 180,000 kr–269,999 kr, Middle II = 270,000 kr–359,999 kr, High I = 360,000 kr–449,999 kr, High II = 450,000 kr–539,999 kr, High III = 540,000 kr+. In Italy Lower I = Under €15,000, Lower II = €15,000–€29,999, Middle I = €30,000–€44,999, Middle II = €45,000–€74,999, High I = €75,000–€124,999, High II = €125,000–€199,999, High III = €200,000+.

**Table A2:** Quartles of Factor Scores by Country-Language Groups.

		USA *n* (%)	Canada English *n* (%)	Canada French *n* (%)	Sweden *n* (%)	Italy *n* (%)
Factor 1 score (Vaccine Effects)						
Q1	239 (33)	95 (19)	113 (23)	269 (28)	199 (20)	915 (25)
Q2	187 (26)	121 (25)	107 (22)	293 (30)	208 (21)	916 (25)
Q3	120 (17)	126 (26)	126 (26)	229 (24)	315 (32)	916 (25)
Q4	181 (25)	151 (31)	146 (30)	174 (18)	264 (27)	916 (25)
Factor 2 score (Vaccine Value)						
Q1	279 (38)	82 (17)	92 (19)	221 (23)	152 (15)	826 (23)
Q2	210 (29)	131 (27)	136 (28)	299 (31)	229 (23)	1005 (27)
Q3	133 (18)	146 (30)	97 (20)	220 (23)	315 (32)	911 (25)
Q4	105 (14)	134 (27)	167 (34)	225 (23)	290 (29)	921 (25)
Factor 3 score (Societal freedoms)						
Q1	154 (21)	126 (26)	114 (23)	285 (30)	230 (23)	909 (25)
Q2	149 (21)	164 (33)	122 (25)	256 (27)	215 (22)	906 (25)
Q3	192 (26)	131 (27)	98 (20)	185 (19)	226 (23)	832 (23)
Q4	232 (32)	72 (15)	158 (32)	239 (25)	315 (32)	1016 (28)

**Table A3:** Model-based estimated odds ratio for likelihood to vaccinate, for contrasts between quartile of factor scores.

Population Logit for	Total		USA		Canada English		Canada French		Sweden		Italy	
	2 + 3 vs. 1	3 vs. 1 + 2	2 + 3 vs. 1	3 vs. 1 + 2	2 + 3 vs. 1	3 vs. 1 + 2	2 + 3 vs. 1	3 vs. 1 + 2	2 + 3 vs. 1	3 vs. 1 + 2	2 + 3 vs. 1	3 vs. 1 + 2
	OR	OR	OR	OR	OR	OR	OR	OR	OR	OR	OR	OR
Factor 1 (Vaccine Effects)												
Q2 vs. Q1	1.4 *	1.0										
Q3 vs. Q1	1.5 ^†^	1.5 ^†^										
Q4 vs. Q1	1.5	2.5 ^†^										
Q3 vs. Q2	1.1	1.6 ^†^										
Q4 vs. Q2	1.1	2.6 ^†^										
Q4 vs. Q3	1.0	1.6 ^†^										
Factor 2 (Vaccine Value)												
Q2 vs. Q1	3.7 ^†^	2.0 ^†^	2.3 ^†^	1.3	2.9 ^†^	1.5	4.8 ^†^	2.6 *	4.4 ^†^	2.4 ^†^	4.8 ^†^	2.6 ^†^
Q3 vs. Q1	12.4 ^†^	8.0 ^†^	7.6 ^†^	3.8 ^†^	17.8 ^†^	9.0 ^†^	15.4 ^†^	7.8 ^†^	8.3 ^†^	14.3 ^†^	16.7 ^†^	8.5 ^†^
Q4 vs. Q1	16.3 ^†^	30.7 ^†^	10.7 ^†^	10.7 ^†^	39.6 ^†^	39.6 ^†^	8.0 ^†^	28.5 ^†^	20.4 ^†^	49.3 ^†^	16.4 ^†^	45.7 ^†^
Q3 vs. Q2	3.3 ^†^	4.0 ^†^	3.3 ^†^	3.0 ^†^	6.2 ^†^	5.8 ^†^	3.2 *	3.0 ^†^	1.9	5.9 ^†^	3.5 ^†^	3.2 ^†^
Q4 vs. Q2	4.4 ^†^	15.3 ^†^	4.6 ^†^	8.5 ^†^	13.9 ^†^	25.6 ^†^	1.7	10.9 ^†^	4.6 *	20.4 ^†^	3.4 *	17.4 ^†^
Q4 vs. Q3	1.3	3.8 ^†^	1.4	2.8 *	2.2	4.4 *	0.5	3.7 ^†^	2.5	3.4 ^†^	1.0	5.4 ^†^
Factor 3 (Societal freedoms)												
Q2 vs. Q1	0.9	0.9	0.8	0.8	1.0	1.0	0.8	0.8	1.2	1.2	0.6 *	0.6 *
Q3 vs. Q1	0.5 ^†^	0.8 *	0.7	0.7	0.3 *	0.7	0.4	1.2	1.1	1.1	0.4 *	0.4 *
Q4 vs. Q1	0.3 ^†^	0.5 ^†^	0.5 *	0.8	0.4 *	0.6	0.4 *	0.6	0.3 ^†^	0.4 ^†^	0.2 ^†^	0.3 ^†^
Q3 vs. Q2	0.6 *	0.9	0.8	0.8	0.3 *	0.7	0.6	1.6	0.9	0.9	0.7	0.7
Q4 vs. Q2	0.4 ^†^	0.6 ^†^	0.7	1.0	0.4 *	0.6	0.5 *	0.7	0.2 ^†^	0.3 ^†^	0.3 ^†^	0.5 ^†^
Q4 vs. Q3	0.6 *	0.6 *	0.8	1.2	1.3	0.9	0.9	0.5 *	0.2 ^†^	0.4 ^†^	0.4 ^†^	0.6 *

* Based on an ordinal regression model under the partial proportional odds assumption for likelihood to vaccinate outcome with predictors for country-language groups, 3 factor score while adjusting for age, gender, education, income, vaccine availability, indicator of prior refusal of taking recommended vaccine, and concerns about COVID-19 scale. 1 Likelihood to vaccinate outcome had three levels: 1 = very unlikely, 2 = unsure, 3 = very likely, and the ordinal regression model estimated logits for levels 2 + 3 vs. 1 and 3 vs. 1 + 2. * *p* < 0.05. † *p* < 0.0001.

## Appendix B. Scale Items

How much do you agree or disagree with the following statements? Note: All items allowed for the following seven possible responses: “Strongly disagree”; “Disagree”; “Somewhat disagree”; “Unsure”; “Somewhat agree”; “Agree” and “Strongly Agree”.

**Table A4:** Scale items.

Item		Strongly Disagree	Disagree	Somewhat Disagree	Unsure	Somewhat Agree	Agree	Strongly Agree
You cannot get COVID-19 from the vaccine itself	misin_1_c19_vax							
There are no toxic ingredients in the vaccine that can harm your health	misin_2_tox_ingred							
The vaccine cannot mess up your DNA	misin_3_DNA							
The vaccine cannot cause infertility	misin_4_infert							
The vaccine cannot cause other diseases	misin_5_other_dis							
The fast production of the vaccine did not compromise its safety	misin_6_fast_prod							
The vaccine is not going to be used by Governments as a tool to limit our civil rights (right of assembly, right of movement, right of religion, etc.)	misin_7_rights							

**Table A5:** Scale items.

Item		Strongly Disagree	Disagree	Somewhat Disagree	Unsure	Somewhat Agree	Agree	Strongly Agree
The vaccine will work in protecting me from getting COVID-19	vax_conf_1_work							
By taking the vaccine I will protect my friends and family from getting COVID-19	vax_conf_2_friends							
Everyone should get the vaccine to achieve herd immunity	vax_conf_3_herd							
Getting COVID-19 is worse than experiencing potential side effects from the vaccine	vax_conf_4_c19worse							
Natural remedies will not protect me from COVID-19	vax_conf_5_natural							

**Table A6:** Scale items.

Item		Strongly Disagree	Disagree	Somewhat Disagree	Unsure	Somewhat Agree	Agree	Strongly Agree
People should be free to decide if they get vaccinated or not with no consequences for their job or personal life	vax_op_1_decide							
People should have the option to choose the vaccine brand they want	vax_op_2_brand							
People should be allowed to live their life with no restrictions once vaccinated	vax_op_3_no_restrict							
Healthcare professionals and scientists with concerns about the vaccine should have opportunities to share their opinions with the public	vax_op_4_hc_share_op							
Everybody should have equal access to the most effective and safe vaccine regardless of income, race, or immigration status	dropped from analysis due to loadings							
There is no elite group that will achieve financial power if people are getting vaccinated	dropped from analysis due to loadings							
There is no microchip with tracking capabilities inserted in the vaccine	vax_op_7_microchip							

## References

[1] World Health Organization Top Ten Threats to Global Health in 2019. https://www.who.int/news-room/spotlight/ten-threats-to-global-health-in-2019.

[2] Rozek L.S., Jones P., Menon A., Hicken A., Apsley S., King E.J. (2021). Understanding Vaccine Hesitancy in the Context of COVID-19: The Role of Trust and Confidence in a Seventeen-Country Survey. Int. J. Public Health.

[3] Lazarus J.V., Wyka K., Rauh L., Rabin K., Ratzan S., Gostin L.O., Larson H.J., El-Mohandes A. (2020). Hesitant or Not? The Association of Age, Gender, and Education with Potential Acceptance of a COVID-19 Vaccine: A Country-level Analysis. J. Health Commun..

[4] Singh K., Lima G., Cha M., Cha C., Kulshrestha J., Ahn Y.-Y., Varol O. (2022). Misinformation, believability, and vaccine acceptance over 40 countries: Takeaways from the initial phase of the COVID-19 infodemic. PLoS ONE.

[5] Sallam M. (2021). COVID-19 vaccine hesitancy worldwide: A concise systematic review of vaccine acceptance rates. Vaccines.

[6] Savoia E., Piltch-Loeb R., Goldberg B., Miller-Idriss C., Hughes B., Montrond A., Kayyem J., Testa M.A. (2021). Predictors of COVID-19 vaccine hesitancy: Socio-demographics, co-morbidity, and past experience of racial discrimination. Vaccines.

[7] Piltch-Loeb R., Silver D.R., Kim Y., Norris H., McNeill E., Abramson D.M. (2022). Determinants of the COVID-19 vaccine hesitancy spectrum. PLoS ONE.

[8] Savoia E., Harriman N.W., Piltch-Loeb R., Bonetti M., Toffolutti V., Testa M.A. (2022). Exploring the Association between Misinformation Endorsement, Opinions on the Government Response, Risk Perception, and COVID-19 Vaccine Hesitancy in the US, Canada, and Italy. Vaccines.

[9] Piltch-Loeb R., Savoia E., Goldberg B., Hughes B., Verhey T., Kayyem J., Miller-Idriss C., Testa M. (2021). Examining the effect of information channel on COVID-19 vaccine acceptance. PLoS ONE.

[10] Piltch-Loeb R., Harriman N.W., Healey J., Bonetti M., Toffolutti V., Testa M.A., Su M., Savoia E. (2021). COVID-19 Vaccine Concerns about Safety, Effectiveness, and Policies in the United States, Canada, Sweden, and Italy among Unvaccinated Individuals. Vaccines.

[11] Centers for Disease Control and Prevention Trends in COVID-19 Vaccine Confidence in the US. https://covid.cdc.gov/covid-data-tracker/#vaccine-confidence.

[12] Griffith J., Marani H., Monkman H. (2021). COVID-19 Vaccine Hesitancy in Canada: Content Analysis of Tweets Using the Theoretical Domains Framework. J. Med. Internet Res..

[13] Moscardino U., Musso P., Inguglia C., Ceccon C., Miconi D., Rousseau C. (2022). Sociodemographic and psychological correlates of COVID-19 vaccine hesitancy and resistance in the young adult population in Italy. Vaccine.

[14] Jang H., Rempel E., Roe I., Adu P., Carenini G., Janjua N.Z. (2022). Tracking Public Attitudes Toward COVID-19 Vaccination on Tweets in Canada: Using Aspect-Based Sentiment Analysis. J. Med. Internet Res..

[15] Fues W.H., Wikman E.B., Sahlin C., Nyaku M., Benĉina G. (2022). Analysis of vaccine messages on social media (Twitter) in Scandinavia. Human Vaccines Immunother..

[16] Rotolo B., Dubé E., Vivion M., MacDonald S.E., Meyer S.B. (2022). Hesitancy towards COVID-19 vaccines on social media in Canada. Vaccine.

[17] Public Health Agency of Canada COVID-19 Vaccine Uptake and Intent: Canadian Community Health Survey (CCHS) Insight. https://www.canada.ca/en/public-health/services/publications/vaccines-immunization/covid-19-vaccine-uptake-intent-canadian-community-health-survey.html#shr-pg0.

[18] Di Giuseppe G., Pelullo C.P., Volgare A.S., Napolitano F., Pavia M. (2022). Parents’ Willingness to Vaccinate Their Children With COVID-19 Vaccine: Results of a Survey in Italy. J. Adolesc. Health.

[19] Ebrahimi O.V., Johnson M.S., Ebling S., Amundsen O.M., Halsøy Ø., Hoffart A., Skjerdingstad N., Johnson S.U. (2021). Risk, Trust, and Flawed Assumptions: Vaccine Hesitancy During the COVID-19 Pandemic. Front. Public Health.

[20] Pollfish. www.pollfish.com.

[21] Government of Canada Coronavirus Disease (COVID-19). https://www.canada.ca/en/public-health/services/diseases/coronavirus-disease-covid-19.html.

[22] How CDC Is Making COVID-19 Vaccine Recommendations. https://www.cdc.gov/coronavirus/2019-ncov/vaccines/recommendations-process.html?CDC_AA_refVal=https%3A%2F%2Fwww.cdc.gov%2Fcoronavirus%2F2019-ncov%2Fvaccines%2Frecommendations.html.

[23] Folkhalsomyndigheten-Public Health Agency of Sweden: Vaccination against COVID-19. https://www.folkhalsomyndigheten.se/the-public-health-agency-of-sweden/communicable-disease-control/covid-19/vaccination-against-covid-19/.

[24] L’epidemiologia per la Sanità Pubblica-Istituto Superiore di Sanità-Piano Nazionale di Vaccinazione COVID-19. https://www.epicentro.iss.it/vaccini/covid-19-piano-vaccinazione.

[25] Rothschild D., Konitzer T. (2018). Random Device Engagement (RDE) with Organic Samples.

[26] Hughes B., Miller-Idriss C., Piltch-Loeb R., Goldberg B., White K., Criezis M., Savoia E. (2021). Development of a Codebook of Online Anti-Vaccination Rhetoric to Manage COVID-19 Vaccine Misinformation. Int. J. Environ. Res. Public Health.

[27] Jodoin M.G., Gierl M.J. (2001). Evaluating type I error and power rates using an effect size measure with the logistic regression procedure for DIF detection. Appl. Meas. Educ..

[28] Petersen M.A., Groenvold M., Bjorner J.B., Aaronson N., Conroy T., Cull A., Fayers P., Hjermstad M., Sprangers M., Sullivan M. (2003). Use of differential item functioning analysis to assess the equivalence of translations of a questionnaire. Qual. Life Res..

[29] Scott N.W., Fayers P.M., Bottomley A., Aaronson N.K., de Graeff A., Groenvold M., Koller M., Petersen M.A. (2006). Sprangers MAG: Comparing translations of the EORTC QLQ-C30 using differential item functioning analyses. Qual. Life Res..

[30] Dziuban C.D., Shirkey E.C. (1974). When is a correlation matrix appropriate for factor analysis?. Psychol. Bull..

[31] Cervantes L., Hazel C.A., Mancini D., Pereira R.I., Podewils L.J., Stella S.A., Durfee J., Barshney A., Steiner J.F. (2022). Perspectives of Latinx Individuals Who Were Unvaccinated and Hospitalized for COVID-19: A Qualitative Study. JAMA Netw. Open.

[32] Kreps S., Dasgupta N., Brownstein J.S., Hswen Y., Kriner D.L. (2021). Public attitudes toward COVID-19 vaccination: The role of vaccine attributes, incentives, and misinformation. NPJ Vaccines.

[33] Palamenghi L., Barello S., Boccia S., Graffigna G. (2020). Mistrust in biomedical research and vaccine hesitancy: The forefront challenge in the battle against COVID-19 in Italy. Eur. J. Epidemiol..

[34] Muric G., Wu Y., Ferrara E. (2021). COVID-19 Vaccine Hesitancy on Social Media: Building a Public Twitter Data Set of Antivaccine Content, Vaccine Misinformation, and Conspiracies. JMIR Public Health Surveill..

[35] Neely S., Eldredge C., Sanders R. (2021). Health Information Seeking Behaviors on Social Media During the COVID-19 Pandemic Among American Social Networking Site Users: Survey Study. J. Med. Internet Res..

[36] Puri N., Coomes E.A., Haghbayan H., Gunaratne K. (2020). Social media and vaccine hesitancy: New updates for the era of COVID-19 and globalized infectious diseases. Hum. Vaccin. Immunother..

[37] Abbasi J. (2022). Widespread Misinformation About Infertility Continues to Create COVID-19 Vaccine Hesitancy. JAMA.

[38] Coronavirus Pandemic (COVID-19). OurWorldInData.org. https://ourworldindata.org/coronavirus.

[39] Ontario Ministry of Health COVID-19 Vaccine Administration. https://www.health.gov.on.ca/en/pro/programs/publichealth/coronavirus/docs/vaccine/COVID-19_vaccine_administration.pdf.

[40] American Journal of Managed Care A Timeline of COVID-19 Vaccine Developments in 2021. https://www.ajmc.com/view/a-timeline-of-covid-19-vaccine-developments-in-2021.

[41] Oliani F., Savoia A., Gallo G., Tiwana N., Letzgus M., Gentiloni F., Piatti A., Chiappa L., Bisesti A., Laquintana D. (2022). Italy’s rollout of COVID-19 vaccinations: The crucial contribution of the first experimental mass vaccination site in Lombardy. Vaccine.

[42] The Local Who Is Eligible for a COVID-19 Vaccine in Your Region of Italy. https://www.thelocal.it/20210506/which-groups-are-eligible-for-a-covid-19-vaccine-in-your-region-of-italy/.

[43] Guay M., Gosselin V., Petit G., Baron G., Gagneur A. (2019). Determinants of vaccine hesitancy in Quebec: A large population-based survey. Hum. Vaccines Immunother..

[44] Lavoie K., Gosselin-Boucher V., Stojanovic J., Gupta S., Gagné M., Joyal-Desmarais K., Séguin K., Gorin S.S., Ribeiro P., Voisard B. (2022). Understanding national trends in COVID-19 vaccine hesitancy in Canada: Results from five sequential cross-sectional representative surveys spanning April 2020-March 2021. BMJ Open.

[45] Bolsen T., Palm R. (2022). Politicization and COVID-19 vaccine resistance in the U.S. Prog. Mol. Biol. Transl. Sci..

[46] The White House Briefing Room Remarks from President Biden on the Fight against COVID-19. https://www.whitehouse.gov/briefing-room/speeches-remarks/2021/12/21/remarks-by-president-biden-on-the-fight-against-covid-19/.

[47] Allin S., Fitzpatrick T., Marchildon G.P., Quesnel-Vallée A. (2022). The federal government and Canada’s COVID-19 responses: From ‘we’re ready, we’re prepared’ to ‘fires are burning’. Health Econ. Policy Law.

[48] Warren G.W., Lofstedt R. (2021). COVID-19 vaccine rollout risk communication strategies in Europe: A rapid response. J. Risk Res..

[49] Stoler J., Klofstad C.A., Enders A.M., Uscinski J.E. (2022). Sociopolitical and psychological correlates of COVID-19 vaccine hesitancy in the United States during summer 2021. Soc. Sci. Med..

